# Roadblocks to Infection Prevention Efforts in Health Care: SARS-CoV-2/COVID-19 Response

**DOI:** 10.1017/dmp.2020.55

**Published:** 2020-03-30

**Authors:** Saskia Popescu

**Affiliations:** HonorHealth, George Mason University, Fairfax, VA

**Keywords:** biopreparedness, health care, hospital preparedness, infection prevention, pandemic

## Abstract

The outbreak of a novel coronavirus, SARS-CoV-2, is challenging international public health and health care efforts. As hospitals work to acquire enough personal protective equipment and brace for potential cases, the role of infection prevention efforts and programs has become increasingly important. Lessons from the 2003 SARS-CoV outbreak in Toronto and 2015 MERS-CoV outbreak in South Korea have unveiled the critical role that hospitals play in outbreaks, especially of novel coronaviruses. Their ability to amplify the spread of disease can rapidly fuel transmission of the disease, and often those failures in infection prevention and general hospital practices contribute to such events. While efforts to enhance infection prevention measures and hospital readiness are underway in the United States, it is important to understand why these programs were not able to maintain continued, sustainable levels of readiness. History has shown that infection prevention programs are primarily responsible for preparing hospitals and responding to biological events but face understaffing and focused efforts defined by administrators. The current US health care system, though, is built upon a series of priorities that often view biopreparedness as a costly endeavor. Awareness of these competing priorities and the challenges that infection prevention programs face when working to maintain biopreparedness is critical in adequately addressing this critical infrastructure in the face of an international outbreak.

The emergence of a novel coronavirus (SARS-CoV-2) causing the disease, COVID-19, in Wuhan, China, in late 2019 is currently testing international public health and preparedness efforts. As the cases of COVID-19 surge past 100 000 and over 1600 have been identified in the United States, the question of capacity and preparedness comes into play. One arm of response lies within health care.

When responding to infectious disease threats, hospitals lean heavily on the expertise and guidance of their infection prevention and control (IPC) programs, which comprise infection preventionists. With backgrounds in nursing, epidemiology, and microbiology, it’s not surprising that IPC would lead such efforts, as it is the intersection of health care and public health. During the 2014 Dallas Ebola cluster, readiness efforts were led by primarily IPC and consumed 80% of their time, and 70% of daily infection prevention duties were not completed during this time.^1^ These IPC outbreak efforts include education, reinforcement of infection prevention standards like isolation and proper usage of personal protective equipment (PPE), and established processes to ensure that the i3 strategy (identify, isolate, and inform) is used and that the health care facilities meet the guidance of the Centers for Disease Control and Prevention and World Health Organization. These strategies are in addition to daily surveillance and reporting requirements, among other duties.

IPC efforts have proven critical in previous coronavirus outbreaks because hospitals can easily act as amplifiers for disease. In 2003, the outbreak of SARS-CoV in Toronto shed light on the ability for hospital transmission to fuel an outbreak. In Canada alone, 43% of the cases were health care workers, and, in phase II of the outbreak and of the cases in Toronto, 72% were a result of nosocomial transmission.^2-4^ In fact, health care transmission rose from 17% in phase I to 88% in phase II of the outbreak. Despite quarantine efforts put into place, the implementation of enhanced infection prevention measures is widely considered to have been the most effective in halting SARS-CoV transmission. The relaxation of these efforts following phase I of the outbreak resulted in the high rate of nosocomial transmission and the second phase of the outbreak.

The MERS-CoV outbreak in South Korea is also a lesson in IPC efforts during coronavirus outbreaks. Of the 186 cases identified in 2015, roughly 91–99% are believed to be from health care transmission^5,6^; 83% of the MERS-CoV transmission events were a result of 5 super-spreaders. This particular outbreak is relevant in that it was, largely in part, due to several factors: overcrowded emergency departments, delays in isolation, infection prevention failures, utilization of family/visitors in the care process, multiple patients per hospital room, and hospital or doctor “shopping.” Prior to a diagnosis, the index case-patient had contact with 600 people across 4 health care facilities. The outbreak is particularly unique in that not only did intrahospital infections occur, but also hospital-to-hospital, which involved 17 hospitals, but ultimately originated from 1.^7,8^ The role of super-spreaders and a health care system using multiple patients per room, coupled with infection prevention failures, is a situation ripe with potential for an explosive outbreak. Prolonged waiting times in crowded emergency rooms with no isolation can also heavily contribute to the spread of respiratory infections like coronavirus.

These historical outbreaks of MERS-CoV and SARS-CoV underscore the role of hospitals in disease transmission and the impact of infection prevention failures. A recent analysis of 138 patients hospitalized with COVID-19 in Wuhan, China, has reiterated these critical lessons in outbreak response.^9^ Of the 138 patients hospitalized, 26% required admission to the intensive care unit, which increases length of stay and is often more resource intensive in terms of health care working staffing and supplies. One particularly concerning finding was that 41% of the hospitalized patients studied were believed to have acquired the disease through human-to-human transmission as a result of hospital exposures. This volume of health-care-associated cases is not wholly unexpected, based off of previous coronavirus outbreaks, but deeply worrisome in that, again, it highlights the potential for hospitals to amplify disease transmission.

In response to these findings and the international efforts to stop the spread of the disease across dozens of countries, infection prevention efforts must be prioritized. Hospitals are the front line for infectious disease response, and their ability to identify, isolate, and inform public health of potential cases is vital to halt transmission. Reinforcing and investing in IPC efforts within hospitals in the United States and globally must happen to combat the outbreak. Unfortunately, to truly use IPC programs for COVID-19 response, it is important to understand why health-care-associated outbreaks and infection prevention failures occur all too often.

Like most public health efforts, attention and resources tend to flood IPC programs during emergent times. The more underlying and systemic issue, though, is that IPC programs are fighting several roadblocks to better strengthen their hospitals’ preparedness for infectious disease threats. First, these programs are notoriously understaffed. A 2018 study by infection preventionists Barles et al. found that, when staffing needs were reviewed, an effective IPC program needed 1.0 full-time, employed infection preventionist for every 69 beds.^10^ Unfortunately, average staffing tends to be at 1 infection preventionist for a minimum of 80–100 beds. More important, though, those IPC efforts tend to be focused on those health-care-associated infection surveillance and reporting requirements that are linked to mandated reporting and Medicare reimbursement. A 2013–2014 study found that these mandated reporting burdens consumed 5 hours a day for infection preventionists.^11^ When put in the context of taking time away from mandated reporting and reimbursement-associated infections, it’s not surprising that hospital administrators would prioritize those efforts over infectious disease events that are often perceived as unlikely. A 2018 Report by the Office Inspector General of the US Department of Health and Human Services assessed hospital readiness to emerging infectious disease threats following the 2013–2016 Ebola outbreak.^12^ The surveyed hospital administrators reported a higher level of preparedness but noted that emergency preparedness personnel often lacked the specialized knowledge required for infectious disease threats. Moreover, administrators noted that it was difficult to integrate procedures specific to emerging infectious diseases, specifically IPC, into emergency plans and that responding to such events was out of the comfort zone for emergency preparedness coordinators. Researchers found that, although a majority of hospital administrators felt more prepared, there were competing priorities to maintaining preparedness and the likelihood of an event was small; 82% of these administrators cited competing priorities for funding and other resources, and 95% reported that competing priorities (eg, increasing focus on active shooter threats and/or desire to not focus on singular threats) reduced the focus on emerging infectious diseases, meaning that not sustaining infectious disease preparedness is a deliberate choice being made by hospital administrators. Because a majority of Ebola preparedness was overseen by the IPC program and infection preventionists, the decision to not invest in infectious disease preparedness can also be seen as a decision to not invest in the public good, that is, IPC. Furthermore, only one-third of respondents could report which tier their hospital fell into in the 2015-established special pathogens tiered hospital framework.^13^

As hospitals in the United States struggle with shortages of PPE and work to prepare for potential cases of COVID-19, it is critical that such efforts not only include IPC, but also seek to invest in biopreparedness. Infection prevention is at the very nexus of biopreparedness in health care, and yet the United States has continuously under-resourced and underused this critical resource. This outbreak should be seen as an opportunity to reinforce hospital preparedness through not only staffing, resources, and surge capacity, but also IPC efforts. In the future, when we evaluate our response to COVID-19 and look to invest in infectious disease readiness and health care preparedness, it is critical that we do not neglect the role of infection prevention programs and the value they bring to biodefense. IPC programs are at the nexus of public health and health care, acting as sentinels for infectious disease threats and a source for tacit knowledge. Recognizing their potential and investing in their utilization serve to combat poor investment in biopreparedness across the health care industry while enhancing U.S. health security.
